# A latent class analysis of international change and continuity in adolescent health and wellbeing: A repeat cross-sectional study

**DOI:** 10.1371/journal.pone.0305124

**Published:** 2024-06-11

**Authors:** Abigail K. Stevely, Laura A. Gray, Hannah Fairbrother, Laura Fenton, Madeleine Henney, Inge Kersbergen, John Holmes

**Affiliations:** 1 Sheffield Addictions Research Group (SARG), Sheffield Centre for Health and Related Research (SCHARR), University of Sheffield, Sheffield, United Kingdom; 2 Health Economics and Decision Science, Sheffield Centre for Health and Related Research (SCHARR), University of Sheffield, Sheffield, United Kingdom; 3 Healthy Lifespan Institute, University of Sheffield, Sheffield, United Kingdom; 4 Faculty of Health, University of Sheffield, Sheffield, United Kingdom; Centre d’Estudis Demogràfics: Centre d’Estudis Demografics, SPAIN

## Abstract

**Background:**

Since the early 2000s, there have been marked trends in adolescent health and wellbeing indicators across Europe, North America and Australia. In particular, there have been substantial declines in youth drinking. We know little about how these trends are underpinned by co-occurring indicators within individuals. This paper aims to analyse change over time in how indicators cluster within individuals and differences in these patterns between five countries with different trends in youth drinking.

**Methods:**

We analysed four waves of repeat cross-sectional survey data from 15-year-olds in England (n = 5942), Italy (n = 5234), the Netherlands (n = 5408), Hungary (n = 5274), and Finland (n = 7446), which were included in the Health Behaviours in School-aged Children (HBSC) study between 2001/02 and 2013/14. We defined clusters of individuals using multigroup latent class analyses which accounts for change over time. The class indicators included health behaviours, attitudes, wellbeing and relationships. We modelled associations between class membership, sex, and family affluence over time.

**Results:**

We identified four classes in all countries: *Overall unhealthy*, *Overall healthy*, *Moderately healthy* and *Substance abstainers with behaviour risk indicators*. The proportion of adolescents in the *Overall unhealthy* class declined between 2001/02 and 2013/14 by between 22.8 percentage points (pp) in England and 3.2pp in Italy. The extent to which indicators of health and wellbeing changed as linked clusters differed across countries, but changes in alcohol consumption, smoking, drug use and sexual activity were typically concurrent. Adolescents with low family affluence were more likely to be in the *Overall unhealthy* class in all years.

**Conclusions:**

Improvements in indicators of adolescent health and well-being are due mainly to concurrent declines in drinking, smoking, sexual activity, and cannabis use, but these declines are not consistently associated with improvements in other domains. They have also not led to reductions in inequalities in indicators of health and well-being.

## Introduction

Health-related behaviours such as substance use, physical activity, and diet are widely recognised as important determinants of life expectancy and other health outcomes [1, 2]. People often establish patterns in these behaviours during adolescence, making it a crucial period with implications for health across the life course [3]. Researchers typically study each behaviour in isolation or add up the number of behaviours engaged in to identify a risk score [1]. However, examining the different combinations of behaviours that adolescents engage in can provide additional insights that can inform health promotion strategies targeting multiple behaviours, identification of groups at high-risk of poor health and wellbeing outcomes and the risk profile of those groups.

Previous research has found strong evidence that clustering analyses can separate adolescents into groups (or clusters) with similar combinations of behaviours and other indicators of health and wellbeing [4–6]. These groups often have good face validity and useful practical implications. These methods are also well suited for reducing the complexity of findings when investigating associations between a wide range of indicators, and classifying broad patterns of behaviour rather than studying single behaviours in isolation [1]. A recent systematic review found that clustering studies often identify two main clusters: first, adolescents with high engagement in substance use, unhealthy diet, low physical activity and sexual activity; and second, adolescents with low engagement in substance use, and patterns of generally health promoting behaviours [1]. The review also found that studies identify a diverse range of additional clusters lying between these groups that differ depending on the population studied, behaviours or measures included and the analytical methods used.

However, there is no evidence that we are aware of on how clusters differ between countries, either cross-sectionally or over time. For example, none of the 41 studies included in the review discussed above analysed comparable data from multiple countries, and only a small number examined change over time [1]. This is problematic as there have been marked international trends in adolescent health-related behaviours since the early 2000s. For example, alcohol consumption, smoking, illicit drug use and sexual activity have all declined in high-income countries across Europe, North America and Australia, albeit to differing degrees [7, 8]. There have also been changes in wider indicators of adolescent health and wellbeing, including an increase in feeling pressured by schoolwork and declines in mental health [9–11]. These trends are likely to have influenced the proportion of adolescents that could be classed as ‘high-risk’ based on these indicators, and the specific combinations of indicators of health and wellbeing seen in those remaining at high-risk.

In this paper, we build on our previous analysis of trends in the clustering of adolescent health and wellbeing indicators in England [12]. Specifically, we aim to describe how the clustering of adolescent health and wellbeing indicators differs across five countries (England, the Netherlands, Italy, Hungary and Finland) and how this changed between 2001/02 and 2013/14. To provide additional insights into health inequalities, we also test whether the relationship between the clusters and two sociodemographic measures, sex and family affluence, changed across the study period. This study is part of a larger project examining the international decline in youth drinking [9, 13]. The five countries we analyse represent different geographic regions within Europe which have different size declines in alcohol use and distinct historical drinking cultures [8, 14].

## Methods

### Data

The Health Behaviours in School-aged Children survey (HBSC) is an international repeat cross-sectional survey [15]. Researchers in each country apply a standardised protocol to collect data from 11, 13 and 15-year-olds on a wide range of health- and wellbeing-related measures. The target sample size is 1500 students per age group, and they are recruited in each country using a clustered sample design that randomly selects schools and then classes within schools. All students within the selected classes then complete the survey at school under exam conditions.

We analysed data from four waves of the HBSC between 2001/02 and 2013/14, focusing on 15-year-olds as the prevalence of alcohol use and other substance use behaviours of interest is very low in the younger age groups. The total sample sizes across all waves in each country were: England (n = 5942), the Netherlands (n = 5408), Italy (n = 5234), Hungary (n = 5274), and Finland (n = 7446).

### Measures

The HBSC survey includes a wide range of indicators of adolescent health and wellbeing and the latent class models used in this analysis are sensitive to the measures used. We therefore conducted a structured online consultation of international academic and non-academic stakeholders to select measures for inclusion in the analyses [see 12 for details]. We considered both traditional behavioural measures (e.g. smoking) and contemporary indicators of wellbeing (e.g. e-media use) [16].

#### Health and wellbeing indicators

S1 Table describes the full questions, response categories and variables used in the analysis, and we briefly summarise this information here. Three dichotomous measures of substance use captured whether respondents consumed alcohol at least once a week, were current smokers and had ever used cannabis. Physical activity was measured using the number of days per week individuals were physically active for sixty minutes, with four categories from low (0–2 days) to high (7 days). Diet was measured using a 28-point scale capturing the frequency of consuming fruit and vegetables separately. We then created a four-category variable reflecting quartiles of the distribution of respondents across the scale. Three measures captured school-related factors. Self-perceived academic achievement was measured using a four-point scale from ‘below average’ to ‘very good’. Feeling pressured by schoolwork was measured using a four-point scale from ‘a lot’ to ‘not at all’. Classmate social support was measured using a 15-point scale based on scores for three items (e.g. Other students accept me as I am). Again, we used quartiles of the distribution for classmate social support to create a four-category variable. Additional dichotomous measures captured whether respondents had ever had sexual intercourse, used e-media daily (including talking on the phone, texting and internet-based communication) and had at least one (step-)parent who is very easy to talk to. Finally, life satisfaction was measured using a ten-point scale from ‘worst possible life for you’ to ‘best possible life for you’, again separated into a four-category variables reflecting quartiles of the distribution across this scale.

#### Socio-demographic predictors

We also used measures of participant sex (female, male) and self-reported family affluence (three-category family affluence scale based on questions assessing car/van/truck ownership, adolescents having their own bedroom, family holidays, and computer ownership [15]) as independent predictors of latent class membership.

### Statistical analysis

We applied the latent class analysis approach used in Stevely et al. [12] to identify unobserved homogenous subgroups of adolescents within each country based on their response patterns for the above indicators of health and wellbeing. The analysis was conducted in three stages using MPLUS version 8.6.

First, we described trends over time and across countries in the indicators of health and wellbeing. Second, since MPLUS removes participants with missing data on independent predictors of class membership from the analysis, we used multiple imputation to impute values for participant sex and family affluence. There was no missing data on participant sex to be imputed, and 3% missing data for family affluence. The imputation generated 50 datasets which included all five countries [17].

The third stage was latent class model selection for each country separately. We selected the number of latent classes to estimate in each model based on model fit statistics, parsimony, and qualitative assessment of class separation using all four pooled cross-sectional survey waves [18]. After examining model fit statistics including AIC (Akaike information criterion), adjusted-BIC (Bayesian information criterion) and entropy, we chose adjusted-BIC as our preferred model fit statistic because there is limited evidence to support using entropy to select number of classes, and the smaller AIC penalty for complexity has been shown to support excess numbers of classes, especially when the sample size is large [19]. Survey year was then included in the models as a grouping variable to allow variation over time in the likelihood of individuals being in each class (class membership probabilities) and the distribution of class members across the categories of each health and wellbeing indicator (conditional response probabilities). We then examined whether models with greater or lesser constraints on the extent of change over time fit the data better by comparing the following options:

1. Fully unconstrained model where both class membership probabilities and conditional response probabilities vary by survey year (i.e. the characteristics of each class and the proportion of individuals within them changes);
2. Semi-constrained model where only class membership probabilities vary by survey year (i.e. the characteristics of each class remain the same but the proportion of individuals within them changes);
3. Fully constrained model where class membership probabilities and conditional response probabilities are constant across survey years (i.e. the characteristics of each class and the proportion of individuals within them does not change).
4. For each of these model options, participant sex and family affluence were included as predictors of class membership. The final stage of the analysis tested whether allowing the relationship between these predictors and class membership to vary over time improved the model fit.

Latent class models used the complex mixture analysis type in MPLUS version 8.6 to analyse the 50 imputed datasets. Models were adjusted to account for the cluster sampling method within schools, and weighted using sample weights [20]. Conditional response probabilities (CRPs) are not provided in MPLUS outputs when analysing imputed datasets so we derived CRPs by applying fixed model parameters from each country-specific final model (estimated using all 50 imputed datasets) to the first imputed dataset.

### Ethical approval

This study was approved by the University of Sheffield’s ethics committee and conforms to the principles embodied in the Declaration of Helsinki. Each country that participated in the HBSC study obtained approval to conduct the survey from their ethics review board or equivalent regulatory body. Participation was voluntary, and informed consent (active or passive) was sought from school administrators, parents and children as per national human subject requirements. We downloaded the dataset on the 26^th^ January 2022, and the authors did not have access to information that could identify individual participants.

## Results

### Descriptive trends in health and wellbeing indicators

**Fig 1:**
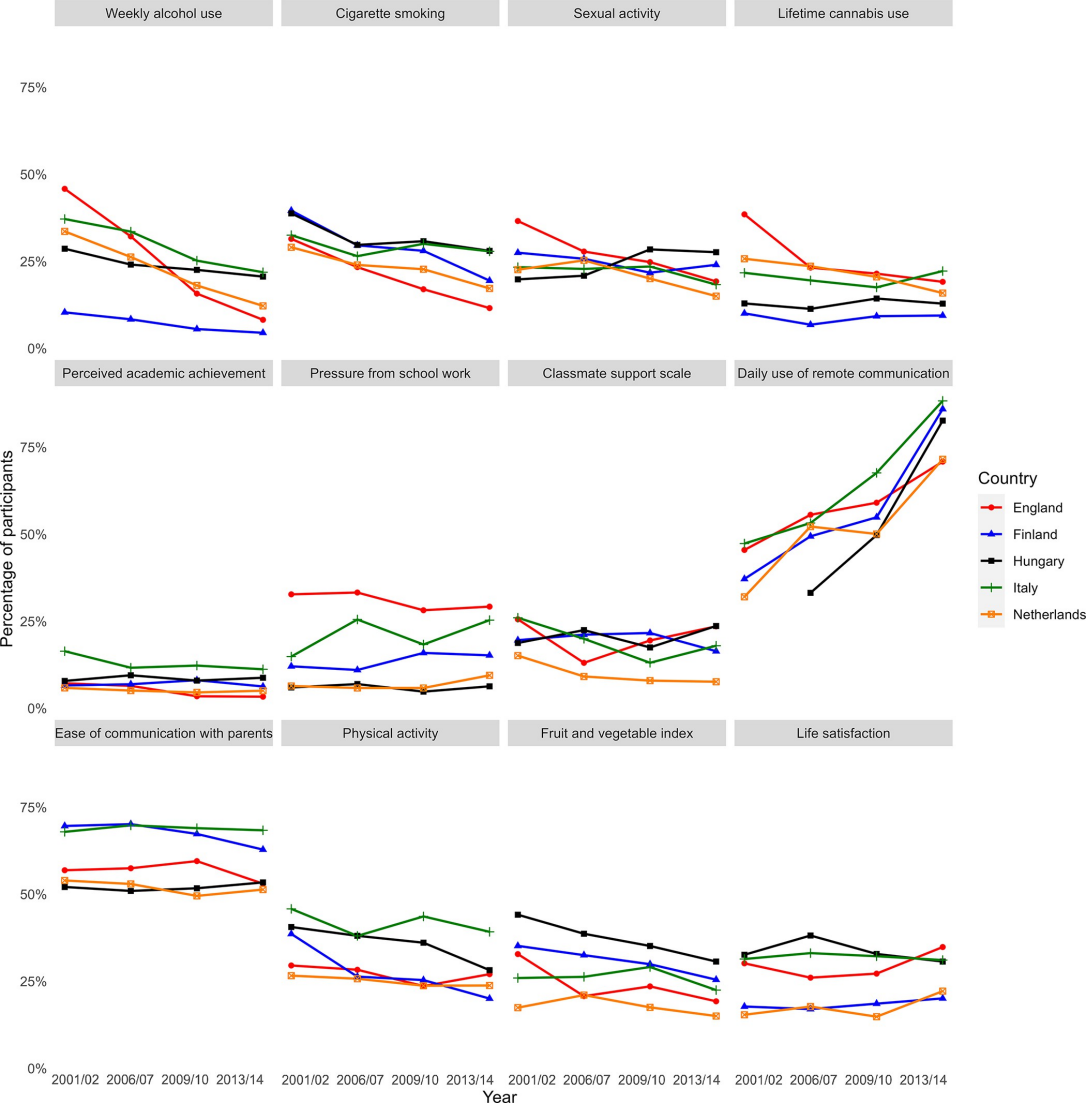
Descriptive trends in health and wellbeing indicators*. *This figure shows the probability that a member of each class in a given year endorses the most risky/unhealthy category for each indicator. For some indicators, there is no clear risky or unhealthy category. Definition of ambiguous variables: Sexual activity—Ever had sexual intercourse; Perceived academic achievement–class teacher thinks that school performance is below average; Pressure from schoolwork–feels a lot of pressure from schoolwork; Classmate support scale–scores in the lowest quartile for support based on how much they agree that A. The students in my class(es) enjoy being together, B. Most of the students in my class(es) are kind and helpful, and C. Other students accept me as I am. All categories chosen are indicated in S1 Table.

Health and wellbeing indicators varied across the five included countries between 2001/02 and 2013/14 (Fig 1, S2 Table). The largest time trends showed decreases in alcohol use, smoking, sexual activity and cannabis use, and increases in use of remote communication. The extent of these trends differed across countries. For example, the prevalence of weekly drinking fell by 38 percentage points (pp) in England (46% to 8%), 22pp in the Netherlands (34% to 12%), 15pp in Italy (37% to 22%), 8pp in Hungary (29% to 21%), and 6pp in Finland (10% to 4%; S2 Table).

### Model fitting

We attempted to fit models with between two and six classes using all four pooled cross-sectional survey waves in each country separately; the models with five or six classes had more model runs that did not converge and the best model solution was often not consistently replicated. Three- and four-class models demonstrated improved model fit compared to two-class models (S3 Table). Qualitative assessment of class separation determined that the four-class model produced meaningful classes in most countries. The adjusted-BIC for Finland was also much lower in the four-class solution relative to the three class solution. We therefore used the four-class solution for the remaining analyses in all five countries to facilitate international comparison. The four-class model solutions had entropy values between 0.62 and 0.74, indicating that the classes are moderately well separated (0.8 or higher would indicate a high degree of class separation). Entropy values were highest for the two-class model solutions in each country. This suggests that although additional classes beyond two improve model fit and result in qualitatively meaningful classes, the additional classes are not as clearly specified.

The fully unconstrained four-class model had the lowest adjusted-BIC for England and Finland, while the semi-constrained model had the lowest adjusted-BIC for the Netherlands, Italy and Hungary (S4 Table). Therefore the final model in England and Finland allowed both class membership and CRPs to vary over time, whereas the final model in the Netherlands, Italy and Hungary allowed class membership probabilities to vary but held CRPs constant. In all countries, the best-fitting model held the relationship between covariates (i.e. sex and family affluence) and class membership constant over time.

### Latent classes in 2013/14

**Fig 2:**
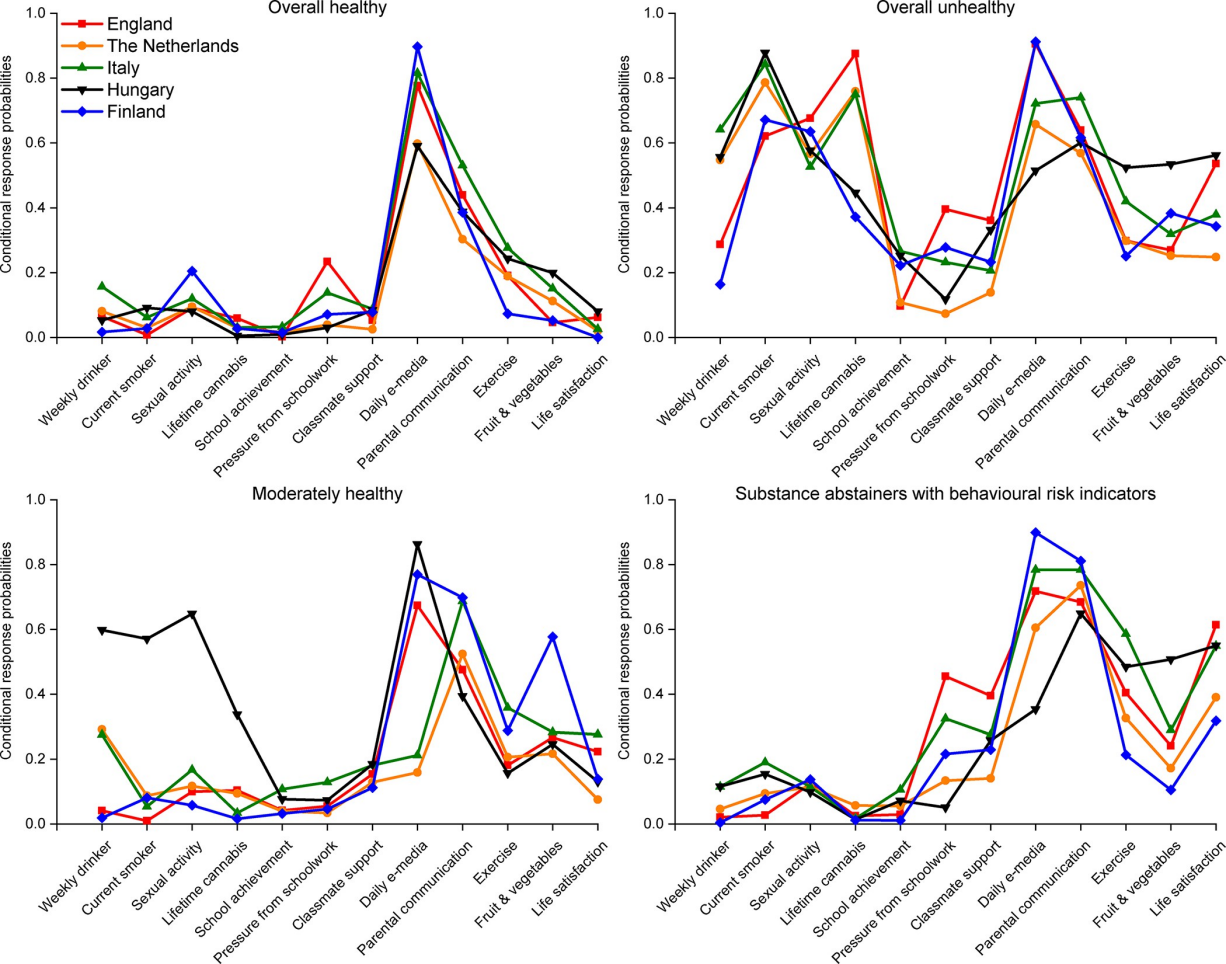
Conditional response probabilities in 2013/14 for each class*. *This figure shows the probability that a member of each class in a given year endorses the most risky/unhealthy category for each indicator. For some indicators, there is no clear risky or unhealthy category. Definition of ambiguous variables: Sexual activity—Ever had sexual intercourse; Perceived academic achievement–class teacher thinks that school performance is below average; Pressure from schoolwork–feels a lot of pressure from schoolwork; Classmate support scale–scores in the lowest quartile for support based on how much they agree that A. The students in my class(es) enjoy being together, B. Most of the students in my class(es) are kind and helpful, and C. Other students accept me as I am. All categories chosen are indicated in S1 Table.

We focus initially on introducing the identified classes and how they differ across countries using the 2013/14 models. Fig 2 shows the CRPs for each class, focusing on the most ‘risky’ or ‘unhealthy’ category of each variable (e.g. weekly drinker, smoker, feels a lot of pressure from school work–see S1 Table for full list). S5 Table provides the full list of CRPs.

The pattern of CRPs in each class is broadly similar across the countries. Individuals in the *Overall unhealthy* class had a high probability of engaging in substance use and sexual activity, engaging in other unhealthy behaviours and reporting other indicators of poor health and wellbeing. Those in the *Substance abstainers with behavioural risk indicators (BRIs)* class were unlikely to engage in substance use and sexual activity, but were more likely to report other indicators of poor health and wellbeing. Those in the *Moderately healthy* class had a lower probability of engaging in substance use or sexual activity than those in the *Substance abstainers with BRIs class*. The exception to this is weekly alcohol use, which was more common in the *Moderately healthy* class in the Netherlands, Italy and particularly Hungary. Individuals in the *Moderately healthy* class also had fewer indicators of poor health and wellbeing overall than the previous two classes. Finally, those in the *Overall healthy* class had the lowest probabilities overall of reporting indicators of poor health or wellbeing.

There were however some notable differences between countries despite the broad similarities. Individuals in the *Moderately healthy* class in Hungary were much more likely to report substance use and sexual activity than those in other countries. Individuals in the *Overall unhealthy* class from England and Finland were much less likely than counterparts in other countries to report weekly drinking. Those in the *Overall unhealthy* class from Finland and Hungary were less likely than their counterparts to report any lifetime cannabis use. Finally, the probability of daily e-media use varied substantially across countries in all classes.

### Change in clustering between 2001/02 and 2013/14

In each country, the proportion of individuals in classes characterised by poorer health and wellbeing decreased across the study period and the proportion in classes characterised by better health and wellbeing increased. However, the classes involved and the extent of this trend differed across countries (Table 1; S5 Table).

**Table 1:** Class membership probabilities over time by country.

Class	Country	Trend^a^	Class membership probabilities (%)			
			2001/02	2005/06	2009/10	2013/14
Overall unhealthy	England	↓↓↓	37.9	23.7	21.4	16.9
	Netherlands	↓↓↓	28.1	26.8	21.5	15.5
	Italy	↓	28.0	23.9	24.8	24.8
	Hungary	↓↓↓	23.1	20.3	18.1	12.8
	Finland	↓	25.9	21.5	20.8	22.1
Substance abstainers with behavioural risk indicators	England	↑	29.1	32.6	26.2	32.6
	Netherlands	↑↑↑	17.6	23.1	21.1	39.3
	Italy	↑↑↑	20.8	23.6	30.3	38.7
	Hungary	↓↓↓	38.0	44.8	33.4	25.4
	Finland	↓↓	27.7	27.6	28.9	21.8
Moderately healthy	England	↑↑	13.8	18.1	26.4	21.3
	Netherlands	↓↓↓	38.7	20.2	22.9	2.6
	Italy	↓↓↓	39.9	33.5	19.4	0.1
	Hungary	↑↑↑	9.9	6.9	15.1	21.9
	Finland	↑↑↑	18.8	21.8	25.9	30.0
Overall healthy	England	↑↑↑	19.1	25.6	26.0	29.2
	Netherlands	↑↑↑	15.7	30.0	34.4	42.7
	Italy	↑↑↑	11.3	19.0	25.4	36.5
	Hungary	↑↑↑	29.0	28.0	33.3	39.9
	Finland	↓	27.6	29.1	24.5	26.1

^a^ ↑/↓ indicates a <5 percentage point (pp) change between 2001/02 and 2013/14, ↑↑/↓↓ includes a 5-10pp change, and ↑↑↑/↓↓↓ indicates a >10pp change.

**Fig 3:**
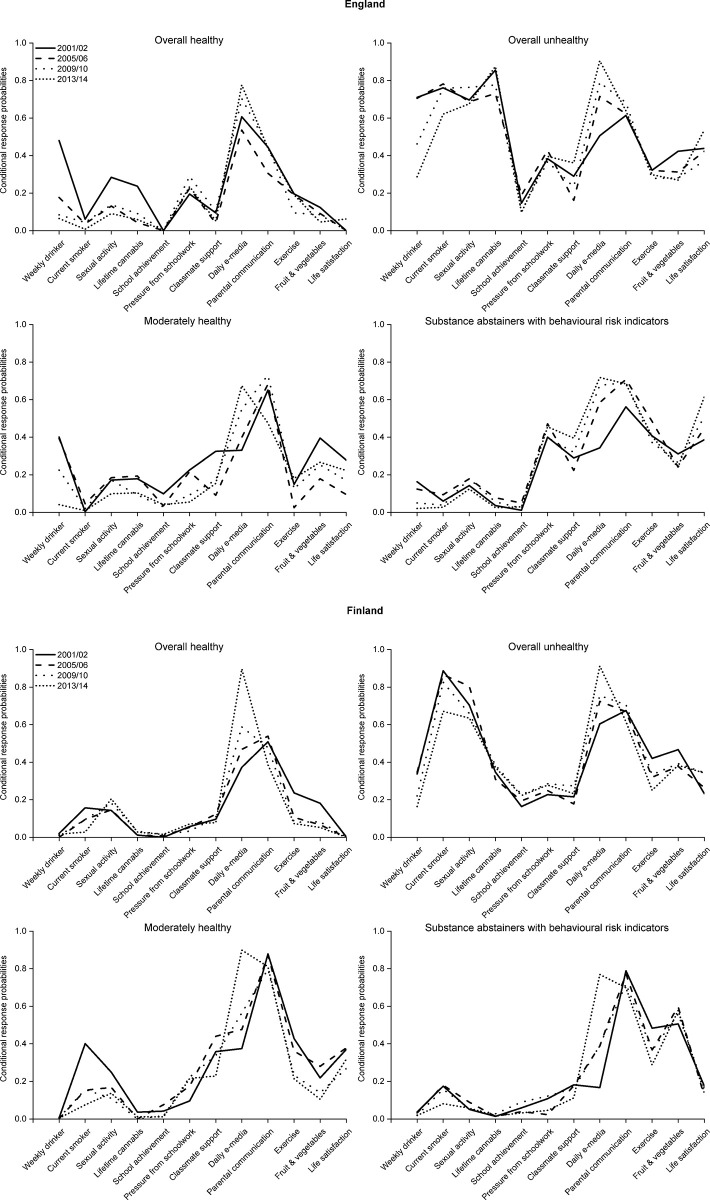
Conditional response probabilities over time for England and Finland*. *This figure shows the probability that a member of each class in a given year endorses the most risky/unhealthy category for each indicator. For some indicators, there is no clear risky or unhealthy category. Definition of ambiguous variables: Sexual activity—Ever had sexual intercourse; Perceived academic achievement–class teacher thinks that school performance is below average; Pressure from schoolwork–feels a lot of pressure from schoolwork; Classmate support scale–scores in the lowest quartile for support based on how much they agree that A. The students in my class(es) enjoy being together, B. Most of the students in my class(es) are kind and helpful, and C. Other students accept me as I am. All categories chosen are indicated in S1 Table.

In England, a 21pp decrease in the proportion of individuals within the *Overall unhealthy* class led to increases in the proportion of individuals in the *Substance abstainers with BRIs* (+3.5pp), *Moderately healthy* (+7.5pp) and *Overall healthy* (+10.1pp) classes. There were also changes in the CRPs within classes in England and Finland, particularly declining likelihoods of weekly drinking and increasing likelihoods of using e-media daily (Fig 3).

In the Netherlands, the proportion of individuals in the *Overall unhealthy* class fell by 12.6pp and the proportion in *Moderately healthy* class fell by 36.1pp. In contrast, there was an increase in the proportions in *Substance abstainers with BRIs* (+21.7pp) and the *Overall healthy* (27.0pp) classes. The changes in Italy followed the same basic patterns.

In Hungary, the proportion of individuals in the *Overall unhealthy* class fell by 10.3pp and the proportion in the *Substance abstainers with BRIs* class also fell by 12.6pp. In contrast, there was an increase in the proportion in the *Moderately healthy* (+12.0pp) and the *Overall healthy* (+10.9pp) classes. These changes reflect improvements across multiple domains of health and wellbeing such as ease of communication with parents and fruit and vegetable consumption, although levels of substance use and sexual activity remained high. Adolescents in Hungary’s atypical *Moderately healthy* class were more likely to report these behaviours than their counterparts in other countries.

Finally, in Finland, there was a smaller decline in the proportion of individuals in the *Overall unhealthy* (-3.8pp) and *Substance abstainers with BRIs* (-5.9pp) classes, an 11.2pp increase in the proportion of individuals in the *Moderately healthy* (+11.2pp) class and little change in the proportion in the *Overall healthy* class (-1.5pp). These smaller changes reflect a smaller decline in substance use and sexual activity than in other countries. There were also changes in CRPs within classes in Finland, particularly declining likelihood of smoking and increasing likelihood of using e-media daily (Fig 3).

### Associations between class membership, sex, and family affluence

We found significant relationships between sex, family affluence and class membership in most countries (Table 2), and these associations were constant over time in all countries. Higher family affluence was associated with increased likelihood of being in the *Overall healthy* class in every country except the Netherlands.

**Table 2:** Class membership by sex and family affluence.

	Odds of being in class vs. *Overall unhealthy* (95% confidence interval)		
	Overall healthy	Moderately healthy	Substance abstainers with behavioural risk indicators
England			
OR for female vs male participant sex	0.82 (0.57,1.19)	0.03 (0.00,7.11)	2.36 (1.59,3.50)*
OR for middle vs low family affluence	2.76 (1.24,6.17)*	2.10 (1.03,4.30)*	1.15 (0.75,1.77)
OR for high vs low family affluence	5.85 (2.55,13.42)*	1.71 (0.80,3.66)	0.73 (0.45,1.20)
The Netherlands			
OR for female vs male participant sex	1.55 (1.04,2.30)*	0.15 (0.05,0.41)*	4.62 (3.13,6.81)*
OR for middle vs low family affluence	0.74 (0.39,1.39)	1.02 (0.37,2.80)	0.61 (0.33,1.12)
OR for high vs low family affluence	0.91 (0.47,1.77)	0.68 (0.23,2.02)	0.44 (0.23,0.81)*
Italy			
OR for female vs male participant sex	1.81 (1.02,3.21)*	0.32 (0.16,0.61)*	6.31 (2.97,13.43)*
OR for middle vs low family affluence	4.07 (1.53,10.84)*	0.96 (0.51,1.79)	0.78 (0.46,1.31)
OR for high vs low family affluence	7.00 (2.40,20.46)*	0.50 (0.25,1.02)	0.54 (0.28,1.05)
Hungary			
OR for female vs male participant sex	1.17 (0.82,1.67)	0.33 (0.14,0.79)*	0.94 (0.65,1.34)
OR for middle vs low family affluence	2.65 (1.86,3.78)*	1.90 (1.07,3.37)*	0.84 (0.62,1.13)
OR for high vs low family affluence	6.31 (3.56,11.17)*	10.27 (5.24,20.12)*	0.65 (0.36,1.18)
Finland			
OR for female vs male participant sex	1.29 (0.89,1.85)	12.83 (7.49,21.98)*	0.24 (0.13,0.44)*
OR for middle vs low family affluence	3.43 (1.87,6.29)*	1.85 (0.96,3.56)	0.94 (0.60,1.47)
OR for high vs low family affluence	6.82 (3.69,12.60)*	2.44 (1.21,4.92)*	0.89 (0.56,1.42)

OR = Odds ratio.

* Indicates odds ratios that do not include 1.

The findings for the relationships between sex and class membership showed greater variation across countries. In general, girls were less likely to be in the *Overall unhealthy* class than boys, and had the highest odds of being in the *Substance abstainers with BRIs* class in England, the Netherlands and Italy. This pattern suggests that girls were less likely to report substance use. The most likely class for boys varied by country, with each class being most likely in at least one country (Table 2).

## Discussion

We identified four classes of adolescents across countries: *Overall unhealthy*, *Overall healthy*, *Moderately healthy*, and *Substance abstainers with behavioural risk indicators*. There was a shift away from the *Overall unhealthy* class in all countries, and the extent of this shift was greatest in countries with larger declines in youth drinking. In all countries aside from Finland the likelihood of adolescents being in the *Overall healthy* class increased over time and this reflected general improvements in health and wellbeing. In particular, levels of alcohol consumption, cigarette smoking, cannabis use and sexual activity declined and appeared strongly clustered within adolescents. Results for the two ‘middle’ classes—*Moderately healthy* and *Substance abstainers with behavioural risk indicators—*were less straightforward to interpret. The characteristics of these classes varied across countries and changes in the proportion of young people within them over time also varied. This suggests the patterns of correlation between indicators of health and well-being differ to some degree across populations. Further, it suggests there is no clear evidence that indicators other than substance use and sexual activity changed in a consistent pattern over time.

The most easily interpretable classes in our models were the *Overall healthy* and *Overall unhealthy* classes. However, a substantial proportion of adolescents do not fit into this two-class model and our findings align with Whitaker et al.’s review [1] by suggesting that there are not straightforwardly identifiable classes that characterise these remaining adolescents. Instead, the additional classes are challenging to interpret across countries or over time even when the indicators and methods used are consistent [1]. For example, differences between countries in the proportion of adolescents in the *Moderately healthy* class over time were hard to interpret as they were sometimes driven by changes in one indicator (e.g. the rise of e-media use over time). In Italy and the Netherlands, adolescents the *Moderately unhealthy* class were unlikely to use e-media daily. Therefore, as e-media use became more common, there was a sharp fall in the proportion of adolescents in this class.

Our findings highlight that a group of adolescents remained in the *Overall unhealthy* class in all five countries in 2013/14 despite the overall improvements in indicators of health and wellbeing and the shift towards the *Overall healthy* class. This group remains at particular risk of poor health outcomes and may require targeted interventions. Adolescents with low family affluence were also much more likely to be in this class in all years, suggesting that overall improvements adolescent health are uneven across the population and may be contributing to the persistence and expansion of health inequalities [21–23].

Key strengths of this study include examining a broad range of health and wellbeing indicators, comparing across five countries and using a large, nationally representative dataset with consistent methods over time and across countries. A limitation is that this clustering has likely changed in the years since 2013/14, particularly during the COVID-19 pandemic. We have not included data from the 2017/18 wave of the HBSC study since this was not available to the research team at the time of analysis. The main trends of interest (e.g. the decline in youth drinking) also occurred largely before 2013/14, and several of the indicators that we use have been changed or removed from the 2017/18 wave [24], hindering meaningful comparisons. Further research using more recent data may provide additional insights into recent changes in adolescent risk profiles. Future studies could also continue to consider explanations for changes in adolescent risk profiles and the persistence of adolescent health inequalities.

## Conclusion

Across five European countries, there are improvements in the overall health and wellbeing of adolescents. These improvements are particularly due to declines in alcohol use, other substance use and sexual activity, which are consistently clustered within adolescents across countries and over time. Changes in other indicators of adolescent health and wellbeing play a less consistent role across countries. These patterns of decline in traditional ‘problem behaviours’ in the absence of wider improvements in health and wellbeing support recent arguments that a general disposition towards risk avoidance and the internalisation of discourse around personal responsibility may be key to understanding the decline in youth alcohol consumption [7, 25–27]. However, despite these declines we found persistent health inequalities [12, 28, 29]. Adolescents with lower family affluence were more likely to be in the *Overall unhealthy* class in all countries except for the Netherlands. As discussed in our previous work, cross-sectoral policy approaches that target the upstream social determinants of health (e.g. increased benefits and financial support for families on low incomes) may be more effective to address this challenge given the clustering of multiple indictors of poor health and wellbeing [12, 28, 29].

## Supporting information

S1 Table Description of variables used to identify classes (indicators) and to predict class membership (covariates), reproduced from Stevely et al. **[12].** *Asterisks are used to indicate the most risky/unhealthy category for each variable. This is used to present results.
(DOCX)

S2 Table Descriptive trends in health and wellbeing indicators. *Using unweighted data from the Health Behaviours in School-aged Children study. Percentages do not sum to 100% because missing responses are not presented.
(DOCX)

S3 Table Selecting number of latent classes using adjusted-BIC. (DOCX)

S4 Table Selecting model specification using adjusted-BIC. (DOCX)

S5 Table Conditional response probabilities. * 1: Overall unhealthy. 2: Substance abstainers with behavioural risk indicators. 3: Moderately healthy. 4: Overall healthy. ** Conditional response probabilities do not change over time for the Netherlands, Italy or Hungary; these results are therefore reported for one year only.
(DOCX)
