# Supplementary material for: A latent class analysis of international change and continuity in adolescent health and wellbeing: A repeat cross-sectional study
Source: PLoS One. 2024 Jun 11;19(6):e0305124. doi: 10.1371/journal.pone.0305124 (PMC11166295; doi:10.1371/journal.pone.0305124)
Supplement: S2 Table — *Using unweighted data from the Health Behaviours in School-aged Children study. Percentages do not sum to 100% because missing responses are not presented. (DOCX) [file pone.0305124.s002.docx]

# **Supplementary Table 2. Descriptive trends in health and wellbeing indicators**

|  | **Percentage of participants by survey year*** | | | |
| --- | --- | --- | --- | --- |
|  | **2001/02** | **2006/07** | **2009/10** | **2013/14** |
| **England** | | | | |
| **Weekly alcohol use** |  |  |  |  |
| Not a weekly drinker | 53% | 67% | 82% | 91% |
| Weekly drinker | 46% | 32% | 16% | 8% |
| **Cigarette smoking** |  |  |  |  |
| Non-smoker | 68% | 76% | 82% | 88% |
| Current smoker | 31% | 23% | 17% | 12% |
| **Sexual activity** |  |  |  |  |
| Never had sexual intercourse | 59% | 69% | 69% | 76% |
| Had sexual intercourse | 37% | 28% | 25% | 19% |
| **Lifetime cannabis use** |  |  |  |  |
| None | 57% | 73% | 77% | 79% |
| Any lifetime use | 39% | 23% | 21% | 19% |
| **Perceived academic achievement** |  |  |  |  |
| Very good | 18% | 20% | 21% | 27% |
| Good | 42% | 43% | 48% | 45% |
| Average | 32% | 26% | 26% | 22% |
| Below average | 7% | 6% | 3% | 3% |
| **Pressure from school work** |  |  |  |  |
| Not at all | 7% | 5% | 7% | 6% |
| A little | 28% | 28% | 33% | 31% |
| Some | 32% | 28% | 30% | 33% |
| A lot | 33% | 33% | 28% | 29% |
| **Classmate support scale** |  |  |  |  |
| High support | 14% | 38% | 19% | 24% |
| - | 22% | 23% | 28% | 24% |
| - | 37% | 21% | 31% | 27% |
| Low support | 26% | 13% | 20% | 24% |
| **Daily use of remote communication** |  |  |  |  |
| Not a daily user | 54% | 42% | 33% | 23% |
| Daily user | 46% | 56% | 59% | 71% |
| **Ease of communication with parents** |  |  |  |  |
| Has one or more parent(s) or step-parent(s) who is/are very easy to talk to | 43% | 42% | 35% | 42% |
| Has no parents or step-parents who are very easy to talk to | 57% | 57% | 60% | 53% |
| **Physical activity** |  |  |  |  |
| High | 16% | 13% | 16% | 13% |
| - | 23% | 24% | 22% | 24% |
| - | 30% | 33% | 33% | 34% |
| Low | 30% | 28% | 24% | 27% |
| **Fruit and vegetable consumption index** |  |  |  |  |
| High | 18% | 31% | 27% | 27% |
| - | 21% | 23% | 22% | 24% |
| - | 28% | 25% | 26% | 29% |
| Low | 33% | 21% | 24% | 19% |
| **Life satisfaction** |  |  |  |  |
| High | 18% | 24% | 18% | 15% |
| - | 25% | 28% | 27% | 25% |
| - | 22% | 21% | 24% | 24% |
| Low | 30% | 26% | 27% | 35% |
| **The Netherlands** | | | | |
| **Weekly alcohol use** |  |  |  |  |
| Not a weekly drinker | 66% | 72% | 81% | 88% |
| Weekly drinker | 34% | 26% | 18% | 12% |
| **Cigarette smoking** |  |  |  |  |
| Non-smoker | 71% | 76% | 76% | 83% |
| Current smoker | 29% | 24% | 23% | 17% |
| **Sexual activity** |  |  |  |  |
| Never had sexual intercourse | 76% | 74% | 76% | 81% |
| Had sexual intercourse | 23% | 25% | 20% | 15% |
| **Lifetime cannabis use** |  |  |  |  |
| None | 74% | 75% | 79% | 84% |
| Any lifetime use | 26% | 24% | 21% | 16% |
| **Perceived academic achievement** |  |  |  |  |
| Very good | 7% | 9% | 10% | 11% |
| Good | 41% | 45% | 48% | 45% |
| Average | 46% | 40% | 34% | 36% |
| Below average | 6% | 5% | 5% | 5% |
| **Pressure from school work** |  |  |  |  |
| Not at all | 24% | 22% | 20% | 16% |
| A little | 53% | 54% | 54% | 48% |
| Some | 16% | 18% | 17% | 24% |
| A lot | 6% | 6% | 6% | 10% |
| **Classmate support scale** |  |  |  |  |
| High support | 23% | 31% | 30% | 33% |
| - | 28% | 32% | 35% | 37% |
| - | 31% | 25% | 23% | 21% |
| Low support | 15% | 9% | 8% | 8% |
| **Daily use of remote communication** |  |  |  |  |
| Not a daily user | 67% | 47% | 49% | 26% |
| Daily user | 32% | 52% | 50% | 72% |
| **Ease of communication with parents** |  |  |  |  |
| Has one or more parent(s) or step-parent(s) who is/are very easy to talk to | 45% | 47% | 50% | 48% |
| Has no parents or step-parents who are very easy to talk to | 54% | 53% | 50% | 51% |
| **Physical activity** |  |  |  |  |
| High | 15% | 17% | 16% | 17% |
| - | 24% | 27% | 31% | 30% |
| - | 32% | 30% | 28% | 28% |
| Low | 27% | 26% | 24% | 24% |
| **Fruit and vegetable consumption index** |  |  |  |  |
| High | 14% | 12% | 11% | 15% |
| - | 32% | 35% | 35% | 36% |
| - | 37% | 31% | 36% | 33% |
| Low | 17% | 21% | 18% | 15% |
| **Life satisfaction** |  |  |  |  |
| High | 29% | 23% | 25% | 21% |
| - | 29% | 34% | 38% | 30% |
| - | 26% | 25% | 21% | 24% |
| Low | 15% | 18% | 15% | 22% |
| **Italy** | | | | |
| **Weekly alcohol use** |  |  |  |  |
| Not a weekly drinker | 63% | 66% | 74% | 78% |
| Weekly drinker | 37% | 34% | 25% | 22% |
| **Cigarette smoking** |  |  |  |  |
| Non-smoker | 67% | 73% | 70% | 72% |
| Current smoker | 33% | 27% | 30% | 28% |
| **Sexual activity** |  |  |  |  |
| Never had sexual intercourse | 76% | 70% | 75% | 71% |
| Had sexual intercourse | 23% | 23% | 24% | 18% |
| **Lifetime cannabis use** |  |  |  |  |
| None | 78% | 74% | 73% | 78% |
| Any lifetime use | 22% | 20% | 18% | 22% |
| **Perceived academic achievement** |  |  |  |  |
| Very good | 13% | 12% | 11% | 11% |
| Good | 27% | 33% | 32% | 33% |
| Average | 43% | 42% | 44% | 44% |
| Below average | 16% | 12% | 12% | 11% |
| **Pressure from school work** |  |  |  |  |
| Not at all | 8% | 7% | 8% | 5% |
| A little | 44% | 33% | 40% | 32% |
| Some | 33% | 32% | 32% | 37% |
| A lot | 15% | 26% | 18% | 25% |
| **Classmate support scale** |  |  |  |  |
| High support | 23% | 32% | 35% | 33% |
| - | 20% | 21% | 25% | 24% |
| - | 30% | 25% | 25% | 24% |
| Low support | 26% | 20% | 13% | 18% |
| **Daily use of remote communication** |  |  |  |  |
| Not a daily user | 52% | 47% | 32% | 11% |
| Daily user | 47% | 53% | 68% | 88% |
| **Ease of communication with parents** |  |  |  |  |
| Has one or more parent(s) or step-parent(s) who is/are very easy to talk to | 32% | 29% | 31% | 31% |
| Has no parents or step-parents who are very easy to talk to | 68% | 70% | 69% | 68% |
| **Physical activity** |  |  |  |  |
| High | 7% | 11% | 8% | 7% |
| - | 15% | 18% | 16% | 20% |
| - | 32% | 31% | 33% | 33% |
| Low | 46% | 38% | 44% | 39% |
| **Fruit and vegetable consumption index** |  |  |  |  |
| High | 20% | 23% | 19% | 23% |
| - | 27% | 27% | 23% | 25% |
| - | 25% | 24% | 29% | 29% |
| Low | 26% | 26% | 29% | 22% |
| **Life satisfaction** |  |  |  |  |
| High | 17% | 21% | 18% | 14% |
| - | 26% | 23% | 24% | 28% |
| - | 25% | 22% | 25% | 26% |
| Low | 31% | 33% | 32% | 31% |
| **Hungary** | | | | |
| **Weekly alcohol use** |  |  |  |  |
| Not a weekly drinker | 71% | 76% | 77% | 79% |
| Weekly drinker | 29% | 24% | 23% | 21% |
| **Cigarette smoking** |  |  |  |  |
| Non-smoker | 61% | 70% | 69% | 71% |
| Current smoker | 39% | 30% | 31% | 28% |
| **Sexual activity** |  |  |  |  |
| Never had sexual intercourse | 80% | 70% | 70% | 69% |
| Had sexual intercourse | 20% | 21% | 28% | 28% |
| **Lifetime cannabis use** |  |  |  |  |
| None | 87% | 82% | 85% | 87% |
| Any lifetime use | 13% | 11% | 14% | 13% |
| **Perceived academic achievement** |  |  |  |  |
| Very good | 7% | 8% | 9% | 10% |
| Good | 28% | 26% | 31% | 30% |
| Average | 57% | 56% | 52% | 50% |
| Below average | 8% | 10% | 8% | 9% |
| **Pressure from school work** |  |  |  |  |
| Not at all | 14% | 19% | 21% | 24% |
| A little | 56% | 53% | 57% | 54% |
| Some | 24% | 20% | 17% | 15% |
| A lot | 6% | 7% | 5% | 6% |
| **Classmate support scale** |  |  |  |  |
| High support | 31% | 29% | 36% | 28% |
| - | 22% | 17% | 20% | 19% |
| - | 27% | 31% | 26% | 29% |
| Low support | 19% | 22% | 18% | 24% |
| **Daily use of remote communication** |  |  |  |  |
| Not a daily user | NA | 66% | 50% | 17% |
| Daily user | NA | 33% | 50% | 83% |
| **Ease of communication with parents** |  |  |  |  |
| Has one or more parent(s) or step-parent(s) who is/are very easy to talk to | 47% | 49% | 48% | 46% |
| Has no parents or step-parents who are very easy to talk to | 52% | 51% | 52% | 53% |
| **Physical activity** |  |  |  |  |
| High | 9% | 14% | 13% | 17% |
| - | 15% | 16% | 16% | 24% |
| - | 34% | 29% | 33% | 29% |
| Low | 41% | 38% | 36% | 28% |
| **Fruit and vegetable consumption index** |  |  |  |  |
| High | 11% | 15% | 15% | 16% |
| - | 16% | 18% | 22% | 23% |
| - | 28% | 28% | 28% | 30% |
| Low | 44% | 39% | 35% | 31% |
| **Life satisfaction** |  |  |  |  |
| High | 19% | 14% | 20% | 21% |
| - | 26% | 23% | 24% | 26% |
| - | 21% | 22% | 22% | 21% |
| Low | 33% | 38% | 33% | 31% |
| **Finland** | | | | |
| **Weekly alcohol use** |  |  |  |  |
| Not a weekly drinker | 89% | 91% | 94% | 94% |
| Weekly drinker | 10% | 8% | 6% | 4% |
| **Cigarette smoking** |  |  |  |  |
| Non-smoker | 60% | 70% | 72% | 80% |
| Current smoker | 40% | 30% | 28% | 19% |
| **Sexual activity** |  |  |  |  |
| Never had sexual intercourse | 70% | 67% | 75% | 73% |
| Had sexual intercourse | 28% | 26% | 22% | 24% |
| **Lifetime cannabis use** |  |  |  |  |
| None | 88% | 88% | 88% | 89% |
| Any lifetime use | 10% | 7% | 9% | 9% |
| **Perceived academic achievement** |  |  |  |  |
| Very good | 15% | 17% | 19% | 22% |
| Good | 34% | 33% | 35% | 37% |
| Average | 43% | 41% | 37% | 34% |
| Below average | 7% | 7% | 8% | 6% |
| **Pressure from school work** |  |  |  |  |
| Not at all | 6% | 6% | 6% | 5% |
| A little | 45% | 44% | 33% | 35% |
| Some | 36% | 37% | 45% | 43% |
| A lot | 12% | 11% | 16% | 15% |
| **Classmate support scale** |  |  |  |  |
| High support | 21% | 18% | 24% | 29% |
| - | 30% | 29% | 28% | 30% |
| - | 27% | 28% | 26% | 24% |
| Low support | 20% | 21% | 22% | 16% |
| **Daily use of remote communication** |  |  |  |  |
| Not a daily user | 62% | 49% | 43% | 13% |
| Daily user | 37% | 49% | 55% | 86% |
| **Ease of communication with parents** |  |  |  |  |
| Has one or more parent(s) or step-parent(s) who is/are very easy to talk to | 30% | 29% | 32% | 37% |
| Has no parents or step-parents who are very easy to talk to | 70% | 70% | 67% | 63% |
| **Physical activity** |  |  |  |  |
| High | 7% | 11% | 14% | 17% |
| - | 19% | 26% | 26% | 27% |
| - | 35% | 31% | 35% | 35% |
| Low | 39% | 26% | 25% | 20% |
| **Fruit and vegetable consumption index** |  |  |  |  |
| High | 12% | 14% | 15% | 14% |
| - | 21% | 21% | 22% | 24% |
| - | 32% | 32% | 33% | 36% |
| Low | 35% | 33% | 30% | 25% |
| **Life satisfaction** |  |  |  |  |
| High | 29% | 30% | 29% | 28% |
| - | 35% | 34% | 34% | 33% |
| - | 17% | 18% | 18% | 19% |
| Low | 18% | 17% | 19% | 20% |

*Using unweighted data from the Health Behaviours in School-aged Children study. Percentages do not sum to 100% because missing responses are not presented
