# Supplementary material for: A latent class analysis of international change and continuity in adolescent health and wellbeing: A repeat cross-sectional study
Source: PLoS One. 2024 Jun 11;19(6):e0305124. doi: 10.1371/journal.pone.0305124 (PMC11166295; doi:10.1371/journal.pone.0305124)
Supplement: S3 Table — (DOCX) [file pone.0305124.s003.docx]

# **Supplementary Table 3. Selecting number of latent classes using adjusted-BIC**

| **Model** | **Adjusted-BIC** | **Change in adjusted-BIC** | **Entropy** |
| --- | --- | --- | --- |
| **England** |  |  |  |
| 2 class | 131053.24 |  | 0.78 |
| 3 class | 130107.11 | -946.13 | 0.66 |
| 4 class | 129688.68 | -418.43 | 0.66 |
| 5 class | 129479.23 | -209.45 | 0.65 |
| 6 class | 129367.11 | -112.12 | 0.62 |
| **The Netherlands** |  |  |  |
| 2 class | 115423.34 |  | 0.78 |
| 3 class | 114526.68 | -896.66 | 0.66 |
| 4 class | 114119.71 | -406.97 | 0.66 |
| 5 class | 113997.36 | -122.35 | 0.66 |
| 6 class | 113896.41 | -100.95 | 0.62 |
| **Italy** |  |  |  |
| 2 class | 114903.49 |  | 0.78 |
| 3 class | 114092.49 | -811.00 | 0.65 |
| 4 class | 113704.87 | -387.62 | 0.74 |
| 5 class | 113524.48 | -180.39 | 0.73 |
| 6 class | 113447.16 | -77.32 | 0.73 |
| **Hungary** |  |  |  |
| 2 class | 111586.36 |  | 0.70 |
| 3 class | 110342.32 | -1244.04 | 0.64 |
| 4 class | 109973.71 | -368.61 | 0.64 |
| 5 class | 109867.59 | -106.12 | 0.67 |
| 6 class | 109787.77 | -79.82 | 0.69 |
| **Finland** |  |  |  |
| 2 class | 155839.02 |  | 0.69 |
| 3 class | 154573.87 | -1265.15 | 0.60 |
| 4 class | 153656.10 | -917.77 | 0.62 |
| 5 class | 153303.45 | -352.65 | 0.62 |
| 6 class | 153134.69 | -168.76 | 0.61 |
