# Supplementary material for: A latent class analysis of international change and continuity in adolescent health and wellbeing: A repeat cross-sectional study
Source: PLoS One. 2024 Jun 11;19(6):e0305124. doi: 10.1371/journal.pone.0305124 (PMC11166295; doi:10.1371/journal.pone.0305124)
Supplement: S4 Table — (DOCX) [file pone.0305124.s004.docx]

**Supplementary Table 4. Selecting model specification using adjusted-BIC**

| **Model** | **Adjusted-BIC** | **Change in adjusted-BIC** |
| --- | --- | --- |
| **England** |  |  |
| Fully constrained | 145766.69 |  |
| Semi-constrained | 145330.95 | -435.75 |
| Fully unconstrained | 145128.84 | -202.11 |
| Fully unconstrained – covariate effect allowed to vary over time | 145268.50 | +139.67 |
| **The Netherlands** |  |  |
| Fully constrained | 129150.98 |  |
| Semi-constrained | 128891.93 | -259.05 |
| Fully unconstrained | 129341.34 | +449.41 |
| Semi-unconstrained – covariate effect allowed to vary over time | 129011.03 | +119.10 |
| **Italy** |  |  |
| Fully constrained | 128191.54 |  |
| Semi-constrained | 128085.54 | -106.00 |
| Fully unconstrained | 128370.32 | +284.79 |
| Semi-unconstrained – covariate effect allowed to vary over time | 128193.55 | +108.01 |
| **Hungary** |  |  |
| Fully constrained | 124441.02 |  |
| Semi-constrained | 124375.18 | -65.84 |
| Fully unconstrained | 124670.67 | +295.49 |
| Semi-unconstrained – covariate effect allowed to vary over time | 124504.49 | +129.31 |
| **Finland** |  |  |
| Fully constrained | 174265.34 |  |
| Semi-constrained | 173951.92 | -313.41 |
| Fully unconstrained | 173738.87 | -213.06 |
| Fully unconstrained – covariate effect allowed to vary over time | 173878.61 | +139.74 |
