# Supplementary material for: A latent class analysis of international change and continuity in adolescent health and wellbeing: A repeat cross-sectional study
Source: PLoS One. 2024 Jun 11;19(6):e0305124. doi: 10.1371/journal.pone.0305124 (PMC11166295; doi:10.1371/journal.pone.0305124)
Supplement: S5 Table — * 1: Overall unhealthy. 2: Substance abstainers with behavioural risk indicators. 3: Moderately healthy. 4: Overall healthy. ** Conditional response probabilities do not change over time for the Netherlands, Italy or Hungary; these results are therefore reported for one year only. (DOCX) [file pone.0305124.s005.docx]

# **Supplementary Table 5. Conditional response probabilities**

|  | **2001/02** | | | | **2005/06** | | | | **2009/10** | | | | **2013/14** | | | |
| --- | --- | --- | --- | --- | --- | --- | --- | --- | --- | --- | --- | --- | --- | --- | --- | --- |
|  | *1** | *2* | *3* | *4* | *1* | *2* | *3* | *4* | *1* | *2* | *3* | *4* | *1* | *2* | *3* | *4* |
| **England** |  |  |  |  |  |  |  |  |  |  |  |  |  |  |  |  |
| **Weekly alcohol use** |  |  |  |  |  |  |  |  |  |  |  |  |  |  |  |  |
| Not weekly | 0.29 | 0.837 | 0.599 | 0.52 | 0.295 | 0.876 | 0.607 | 0.823 | 0.538 | 0.95 | 0.775 | 0.916 | 0.713 | 0.979 | 0.959 | 0.935 |
| Weekly | 0.71 | 0.163 | 0.401 | 0.48 | 0.705 | 0.124 | 0.393 | 0.177 | 0.462 | 0.05 | 0.225 | 0.084 | 0.287 | 0.021 | 0.041 | 0.065 |
| **Cigarette smoking** |  |  |  |  |  |  |  |  |  |  |  |  |  |  |  |  |
| Non-smoker | 0.239 | 0.94 | 0.997 | 0.939 | 0.218 | 0.906 | 0.954 | 0.963 | 0.241 | 0.962 | 0.98 | 0.956 | 0.379 | 0.973 | 0.99 | 0.992 |
| Smoker | 0.761 | 0.06 | 0.003 | 0.061 | 0.782 | 0.094 | 0.046 | 0.037 | 0.759 | 0.038 | 0.02 | 0.044 | 0.621 | 0.027 | 0.01 | 0.008 |
| **Sexual activity** |  |  |  |  |  |  |  |  |  |  |  |  |  |  |  |  |
| Never | 0.304 | 0.857 | 0.828 | 0.716 | 0.314 | 0.821 | 0.815 | 0.869 | 0.236 | 0.82 | 0.828 | 0.862 | 0.324 | 0.876 | 0.9 | 0.909 |
| Had intercourse | 0.696 | 0.143 | 0.172 | 0.284 | 0.686 | 0.179 | 0.185 | 0.131 | 0.764 | 0.18 | 0.172 | 0.138 | 0.676 | 0.124 | 0.1 | 0.091 |
| **Lifetime cannabis use** |  |  |  |  |  |  |  |  |  |  |  |  |  |  |  |  |
| Never | 0.144 | 0.964 | 0.821 | 0.763 | 0.268 | 0.922 | 0.806 | 0.957 | 0.224 | 0.944 | 0.907 | 0.909 | 0.125 | 0.975 | 0.896 | 0.941 |
| Any use | 0.856 | 0.036 | 0.179 | 0.237 | 0.732 | 0.078 | 0.194 | 0.043 | 0.776 | 0.056 | 0.093 | 0.091 | 0.875 | 0.025 | 0.104 | 0.059 |
| **Perceived academic achievement** |  |  |  |  |  |  |  |  |  |  |  |  |  |  |  |  |
| Very good | 0.07 | 0.299 | 0.028 | 0.346 | 0.09 | 0.159 | 0.111 | 0.461 | 0.116 | 0.175 | 0.114 | 0.414 | 0.166 | 0.225 | 0.124 | 0.498 |
| Good | 0.341 | 0.456 | 0.422 | 0.537 | 0.307 | 0.487 | 0.543 | 0.478 | 0.411 | 0.509 | 0.554 | 0.46 | 0.426 | 0.496 | 0.458 | 0.457 |
| Average | 0.442 | 0.234 | 0.451 | 0.117 | 0.414 | 0.304 | 0.311 | 0.061 | 0.369 | 0.294 | 0.304 | 0.127 | 0.311 | 0.25 | 0.376 | 0.044 |
| Below average | 0.146 | 0.011 | 0.099 | 0 | 0.189 | 0.05 | 0.035 | 0 | 0.103 | 0.022 | 0.029 | 0 | 0.097 | 0.029 | 0.042 | 0.002 |
| **Pressure from school work** |  |  |  |  |  |  |  |  |  |  |  |  |  |  |  |  |
| Not at all | 0.095 | 0.044 | 0.074 | 0.07 | 0.061 | 0.015 | 0.062 | 0.097 | 0.061 | 0.024 | 0.117 | 0.079 | 0.069 | 0.022 | 0.08 | 0.089 |
| A little | 0.222 | 0.222 | 0.414 | 0.382 | 0.238 | 0.257 | 0.29 | 0.397 | 0.274 | 0.284 | 0.479 | 0.326 | 0.27 | 0.211 | 0.499 | 0.321 |
| Some | 0.299 | 0.334 | 0.286 | 0.353 | 0.273 | 0.257 | 0.431 | 0.28 | 0.297 | 0.295 | 0.311 | 0.306 | 0.265 | 0.312 | 0.366 | 0.356 |
| A lot | 0.383 | 0.4 | 0.226 | 0.195 | 0.428 | 0.471 | 0.217 | 0.226 | 0.368 | 0.397 | 0.093 | 0.289 | 0.396 | 0.455 | 0.055 | 0.234 |
| **Classmate support scale** |  |  |  |  |  |  |  |  |  |  |  |  |  |  |  |  |
| High support | 0.13 | 0.135 | 0.04 | 0.258 | 0.367 | 0.278 | 0.272 | 0.673 | 0.191 | 0.111 | 0.113 | 0.316 | 0.161 | 0.078 | 0.215 | 0.503 |
| - | 0.223 | 0.193 | 0.141 | 0.34 | 0.225 | 0.252 | 0.322 | 0.173 | 0.262 | 0.216 | 0.296 | 0.382 | 0.205 | 0.212 | 0.284 | 0.254 |
| - | 0.357 | 0.383 | 0.494 | 0.306 | 0.246 | 0.247 | 0.315 | 0.115 | 0.315 | 0.353 | 0.42 | 0.201 | 0.273 | 0.315 | 0.347 | 0.19 |
| Low support | 0.29 | 0.289 | 0.325 | 0.095 | 0.162 | 0.224 | 0.091 | 0.039 | 0.233 | 0.319 | 0.172 | 0.102 | 0.361 | 0.395 | 0.154 | 0.053 |
| **Daily use of remote communication** |  |  |  |  |  |  |  |  |  |  |  |  |  |  |  |  |
| No daily use | 0.494 | 0.657 | 0.67 | 0.393 | 0.285 | 0.416 | 0.602 | 0.464 | 0.211 | 0.324 | 0.452 | 0.296 | 0.095 | 0.282 | 0.326 | 0.224 |
| Daily use | 0.506 | 0.343 | 0.33 | 0.607 | 0.715 | 0.584 | 0.398 | 0.536 | 0.789 | 0.676 | 0.548 | 0.704 | 0.905 | 0.718 | 0.674 | 0.776 |
| **Ease of communication with parents** |  |  |  |  |  |  |  |  |  |  |  |  |  |  |  |  |
| One easy to talk to | 0.386 | 0.438 | 0.349 | 0.555 | 0.377 | 0.292 | 0.318 | 0.694 | 0.317 | 0.309 | 0.269 | 0.552 | 0.361 | 0.316 | 0.525 | 0.56 |
| None easy to talk to | 0.614 | 0.562 | 0.651 | 0.445 | 0.623 | 0.708 | 0.682 | 0.306 | 0.683 | 0.691 | 0.731 | 0.448 | 0.639 | 0.684 | 0.475 | 0.44 |
| **Physical activity** |  |  |  |  |  |  |  |  |  |  |  |  |  |  |  |  |
| High | 0.169 | 0.08 | 0.207 | 0.247 | 0.176 | 0.049 | 0.207 | 0.155 | 0.199 | 0.104 | 0.262 | 0.164 | 0.16 | 0.049 | 0.174 | 0.196 |
| - | 0.204 | 0.192 | 0.304 | 0.313 | 0.219 | 0.116 | 0.381 | 0.31 | 0.232 | 0.18 | 0.261 | 0.333 | 0.195 | 0.182 | 0.319 | 0.285 |
| - | 0.306 | 0.321 | 0.34 | 0.245 | 0.284 | 0.351 | 0.387 | 0.343 | 0.291 | 0.344 | 0.351 | 0.408 | 0.347 | 0.365 | 0.327 | 0.328 |
| Low | 0.322 | 0.407 | 0.149 | 0.196 | 0.321 | 0.484 | 0.025 | 0.191 | 0.279 | 0.372 | 0.126 | 0.095 | 0.298 | 0.404 | 0.181 | 0.191 |
| **Fruit and vegetable consumption index** |  |  |  |  |  |  |  |  |  |  |  |  |  |  |  |  |
| High consumption | 0.108 | 0.198 | 0.066 | 0.351 | 0.269 | 0.253 | 0.214 | 0.477 | 0.21 | 0.274 | 0.139 | 0.488 | 0.245 | 0.214 | 0.122 | 0.467 |
| - | 0.182 | 0.241 | 0.157 | 0.269 | 0.209 | 0.225 | 0.286 | 0.225 | 0.224 | 0.245 | 0.212 | 0.227 | 0.186 | 0.256 | 0.18 | 0.3 |
| - | 0.287 | 0.251 | 0.382 | 0.257 | 0.209 | 0.28 | 0.321 | 0.208 | 0.284 | 0.216 | 0.396 | 0.2 | 0.3 | 0.289 | 0.431 | 0.188 |
| Low consumption | 0.423 | 0.311 | 0.395 | 0.124 | 0.312 | 0.242 | 0.179 | 0.091 | 0.281 | 0.265 | 0.253 | 0.086 | 0.27 | 0.241 | 0.267 | 0.046 |
| **Life satisfaction** |  |  |  |  |  |  |  |  |  |  |  |  |  |  |  |  |
| High | 0.094 | 0.16 | 0.141 | 0.428 | 0.124 | 0.11 | 0.273 | 0.477 | 0.175 | 0.03 | 0.151 | 0.422 | 0.084 | 0.038 | 0.112 | 0.339 |
| - | 0.226 | 0.232 | 0.253 | 0.399 | 0.236 | 0.22 | 0.334 | 0.383 | 0.207 | 0.196 | 0.324 | 0.439 | 0.161 | 0.117 | 0.253 | 0.461 |
| - | 0.242 | 0.223 | 0.327 | 0.173 | 0.218 | 0.224 | 0.298 | 0.138 | 0.245 | 0.266 | 0.356 | 0.139 | 0.219 | 0.231 | 0.412 | 0.139 |
| Low | 0.438 | 0.386 | 0.278 | 0 | 0.423 | 0.446 | 0.095 | 0.001 | 0.372 | 0.508 | 0.17 | 0 | 0.536 | 0.614 | 0.223 | 0.062 |
| **The Netherlands**** |  |  |  |  |  |  |  |  |  |  |  |  |  |  |  |  |
| **Weekly alcohol use** |  |  |  |  |  |  |  |  |  |  |  |  |  |  |  |  |
| Not weekly | 0.453 | 0.954 | 0.709 | 0.919 |  |  |  |  |  |  |  |  |  |  |  |  |
| Weekly | 0.547 | 0.046 | 0.291 | 0.081 |  |  |  |  |  |  |  |  |  |  |  |  |
| **Cigarette smoking** |  |  |  |  |  |  |  |  |  |  |  |  |  |  |  |  |
| Non-smoker | 0.214 | 0.906 | 0.913 | 0.97 |  |  |  |  |  |  |  |  |  |  |  |  |
| Smoker | 0.786 | 0.094 | 0.087 | 0.03 |  |  |  |  |  |  |  |  |  |  |  |  |
| **Sexual activity** |  |  |  |  |  |  |  |  |  |  |  |  |  |  |  |  |
| Never | 0.434 | 0.886 | 0.883 | 0.905 |  |  |  |  |  |  |  |  |  |  |  |  |
| Had intercourse | 0.566 | 0.114 | 0.117 | 0.095 |  |  |  |  |  |  |  |  |  |  |  |  |
| **Lifetime cannabis use** |  |  |  |  |  |  |  |  |  |  |  |  |  |  |  |  |
| Never | 0.241 | 0.943 | 0.906 | 0.971 |  |  |  |  |  |  |  |  |  |  |  |  |
| Any use | 0.759 | 0.057 | 0.094 | 0.029 |  |  |  |  |  |  |  |  |  |  |  |  |
| **Perceived academic achievement** |  |  |  |  |  |  |  |  |  |  |  |  |  |  |  |  |
| Very good | 0.068 | 0.035 | 0.062 | 0.186 |  |  |  |  |  |  |  |  |  |  |  |  |
| Good | 0.366 | 0.415 | 0.445 | 0.567 |  |  |  |  |  |  |  |  |  |  |  |  |
| Average | 0.458 | 0.494 | 0.453 | 0.232 |  |  |  |  |  |  |  |  |  |  |  |  |
| Below average | 0.108 | 0.056 | 0.04 | 0.016 |  |  |  |  |  |  |  |  |  |  |  |  |
| **Pressure from school work** |  |  |  |  |  |  |  |  |  |  |  |  |  |  |  |  |
| Not at all | 0.268 | 0.075 | 0.263 | 0.235 |  |  |  |  |  |  |  |  |  |  |  |  |
| A little | 0.476 | 0.478 | 0.608 | 0.567 |  |  |  |  |  |  |  |  |  |  |  |  |
| Some | 0.182 | 0.313 | 0.095 | 0.159 |  |  |  |  |  |  |  |  |  |  |  |  |
| A lot | 0.073 | 0.134 | 0.034 | 0.039 |  |  |  |  |  |  |  |  |  |  |  |  |
| **Classmate support scale** |  |  |  |  |  |  |  |  |  |  |  |  |  |  |  |  |
| High support | 0.294 | 0.191 | 0.187 | 0.476 |  |  |  |  |  |  |  |  |  |  |  |  |
| - | 0.296 | 0.342 | 0.367 | 0.349 |  |  |  |  |  |  |  |  |  |  |  |  |
| - | 0.271 | 0.326 | 0.317 | 0.15 |  |  |  |  |  |  |  |  |  |  |  |  |
| Low support | 0.139 | 0.141 | 0.129 | 0.025 |  |  |  |  |  |  |  |  |  |  |  |  |
| **Daily use of remote communication** |  |  |  |  |  |  |  |  |  |  |  |  |  |  |  |  |
| No daily use | 0.343 | 0.395 | 0.841 | 0.403 |  |  |  |  |  |  |  |  |  |  |  |  |
| Daily use | 0.657 | 0.605 | 0.159 | 0.597 |  |  |  |  |  |  |  |  |  |  |  |  |
| **Ease of communication with parents** |  |  |  |  |  |  |  |  |  |  |  |  |  |  |  |  |
| One easy to talk to | 0.432 | 0.264 | 0.476 | 0.697 |  |  |  |  |  |  |  |  |  |  |  |  |
| None easy to talk to | 0.568 | 0.736 | 0.524 | 0.303 |  |  |  |  |  |  |  |  |  |  |  |  |
| **Physical activity** |  |  |  |  |  |  |  |  |  |  |  |  |  |  |  |  |
| High | 0.205 | 0.114 | 0.143 | 0.186 |  |  |  |  |  |  |  |  |  |  |  |  |
| - | 0.222 | 0.283 | 0.297 | 0.32 |  |  |  |  |  |  |  |  |  |  |  |  |
| - | 0.274 | 0.277 | 0.354 | 0.305 |  |  |  |  |  |  |  |  |  |  |  |  |
| Low | 0.298 | 0.326 | 0.206 | 0.189 |  |  |  |  |  |  |  |  |  |  |  |  |
| **Fruit and vegetable consumption index** |  |  |  |  |  |  |  |  |  |  |  |  |  |  |  |  |
| High consumption | 0.126 | 0.133 | 0.084 | 0.165 |  |  |  |  |  |  |  |  |  |  |  |  |
| - | 0.273 | 0.367 | 0.251 | 0.451 |  |  |  |  |  |  |  |  |  |  |  |  |
| - | 0.348 | 0.329 | 0.448 | 0.272 |  |  |  |  |  |  |  |  |  |  |  |  |
| Low consumption | 0.252 | 0.172 | 0.217 | 0.112 |  |  |  |  |  |  |  |  |  |  |  |  |
| **Life satisfaction** |  |  |  |  |  |  |  |  |  |  |  |  |  |  |  |  |
| High | 0.186 | 0.015 | 0.302 | 0.441 |  |  |  |  |  |  |  |  |  |  |  |  |
| - | 0.303 | 0.224 | 0.383 | 0.422 |  |  |  |  |  |  |  |  |  |  |  |  |
| - | 0.262 | 0.371 | 0.24 | 0.117 |  |  |  |  |  |  |  |  |  |  |  |  |
| Low | 0.248 | 0.39 | 0.075 | 0.02 |  |  |  |  |  |  |  |  |  |  |  |  |
| **Italy**** |  |  |  |  |  |  |  |  |  |  |  |  |  |  |  |  |
| **Weekly alcohol use** |  |  |  |  |  |  |  |  |  |  |  |  |  |  |  |  |
| Not weekly | 0.359 | 0.885 | 0.725 | 0.843 |  |  |  |  |  |  |  |  |  |  |  |  |
| Weekly | 0.641 | 0.115 | 0.275 | 0.157 |  |  |  |  |  |  |  |  |  |  |  |  |
| **Cigarette smoking** |  |  |  |  |  |  |  |  |  |  |  |  |  |  |  |  |
| Non-smoker | 0.157 | 0.81 | 0.946 | 0.938 |  |  |  |  |  |  |  |  |  |  |  |  |
| Smoker | 0.843 | 0.19 | 0.054 | 0.062 |  |  |  |  |  |  |  |  |  |  |  |  |
| **Sexual activity** |  |  |  |  |  |  |  |  |  |  |  |  |  |  |  |  |
| Never | 0.474 | 0.885 | 0.833 | 0.88 |  |  |  |  |  |  |  |  |  |  |  |  |
| Had intercourse | 0.526 | 0.115 | 0.167 | 0.12 |  |  |  |  |  |  |  |  |  |  |  |  |
| **Lifetime cannabis use** |  |  |  |  |  |  |  |  |  |  |  |  |  |  |  |  |
| Never | 0.25 | 0.983 | 0.966 | 0.969 |  |  |  |  |  |  |  |  |  |  |  |  |
| Any use | 0.75 | 0.017 | 0.034 | 0.031 |  |  |  |  |  |  |  |  |  |  |  |  |
| **Perceived academic achievement** |  |  |  |  |  |  |  |  |  |  |  |  |  |  |  |  |
| Very good | 0.053 | 0.105 | 0.13 | 0.193 |  |  |  |  |  |  |  |  |  |  |  |  |
| Good | 0.19 | 0.304 | 0.334 | 0.437 |  |  |  |  |  |  |  |  |  |  |  |  |
| Average | 0.491 | 0.484 | 0.43 | 0.337 |  |  |  |  |  |  |  |  |  |  |  |  |
| Below average | 0.266 | 0.106 | 0.107 | 0.033 |  |  |  |  |  |  |  |  |  |  |  |  |
| **Pressure from school work** |  |  |  |  |  |  |  |  |  |  |  |  |  |  |  |  |
| Not at all | 0.097 | 0.017 | 0.103 | 0.078 |  |  |  |  |  |  |  |  |  |  |  |  |
| A little | 0.353 | 0.273 | 0.464 | 0.443 |  |  |  |  |  |  |  |  |  |  |  |  |
| Some | 0.318 | 0.385 | 0.304 | 0.341 |  |  |  |  |  |  |  |  |  |  |  |  |
| A lot | 0.232 | 0.325 | 0.129 | 0.138 |  |  |  |  |  |  |  |  |  |  |  |  |
| **Classmate support scale** |  |  |  |  |  |  |  |  |  |  |  |  |  |  |  |  |
| High support | 0.301 | 0.223 | 0.284 | 0.476 |  |  |  |  |  |  |  |  |  |  |  |  |
| - | 0.226 | 0.197 | 0.252 | 0.253 |  |  |  |  |  |  |  |  |  |  |  |  |
| - | 0.267 | 0.306 | 0.282 | 0.183 |  |  |  |  |  |  |  |  |  |  |  |  |
| Low support | 0.206 | 0.275 | 0.181 | 0.088 |  |  |  |  |  |  |  |  |  |  |  |  |
| **Daily use of remote communication** |  |  |  |  |  |  |  |  |  |  |  |  |  |  |  |  |
| No daily use | 0.279 | 0.216 | 0.788 | 0.185 |  |  |  |  |  |  |  |  |  |  |  |  |
| Daily use | 0.721 | 0.784 | 0.212 | 0.815 |  |  |  |  |  |  |  |  |  |  |  |  |
| **Ease of communication with parents** |  |  |  |  |  |  |  |  |  |  |  |  |  |  |  |  |
| One easy to talk to | 0.26 | 0.216 | 0.313 | 0.47 |  |  |  |  |  |  |  |  |  |  |  |  |
| None easy to talk to | 0.74 | 0.784 | 0.687 | 0.53 |  |  |  |  |  |  |  |  |  |  |  |  |
| **Physical activity** |  |  |  |  |  |  |  |  |  |  |  |  |  |  |  |  |
| High | 0.094 | 0.038 | 0.103 | 0.117 |  |  |  |  |  |  |  |  |  |  |  |  |
| - | 0.173 | 0.101 | 0.185 | 0.241 |  |  |  |  |  |  |  |  |  |  |  |  |
| - | 0.312 | 0.274 | 0.354 | 0.365 |  |  |  |  |  |  |  |  |  |  |  |  |
| Low | 0.42 | 0.586 | 0.358 | 0.277 |  |  |  |  |  |  |  |  |  |  |  |  |
| **Fruit and vegetable consumption index** |  |  |  |  |  |  |  |  |  |  |  |  |  |  |  |  |
| High consumption | 0.171 | 0.188 | 0.171 | 0.32 |  |  |  |  |  |  |  |  |  |  |  |  |
| - | 0.243 | 0.248 | 0.265 | 0.267 |  |  |  |  |  |  |  |  |  |  |  |  |
| - | 0.267 | 0.275 | 0.281 | 0.262 |  |  |  |  |  |  |  |  |  |  |  |  |
| Low consumption | 0.319 | 0.289 | 0.283 | 0.151 |  |  |  |  |  |  |  |  |  |  |  |  |
| **Life satisfaction** |  |  |  |  |  |  |  |  |  |  |  |  |  |  |  |  |
| High | 0.159 | 0.025 | 0.202 | 0.366 |  |  |  |  |  |  |  |  |  |  |  |  |
| - | 0.213 | 0.155 | 0.276 | 0.397 |  |  |  |  |  |  |  |  |  |  |  |  |
| - | 0.248 | 0.271 | 0.246 | 0.211 |  |  |  |  |  |  |  |  |  |  |  |  |
| Low | 0.379 | 0.549 | 0.276 | 0.026 |  |  |  |  |  |  |  |  |  |  |  |  |
| **Hungary**** |  |  |  |  |  |  |  |  |  |  |  |  |  |  |  |  |
| **Weekly alcohol use** |  |  |  |  |  |  |  |  |  |  |  |  |  |  |  |  |
| Not weekly | 0.444 | 0.884 | 0.402 | 0.947 |  |  |  |  |  |  |  |  |  |  |  |  |
| Weekly | 0.556 | 0.116 | 0.598 | 0.053 |  |  |  |  |  |  |  |  |  |  |  |  |
| **Cigarette smoking** |  |  |  |  |  |  |  |  |  |  |  |  |  |  |  |  |
| Non-smoker | 0.122 | 0.846 | 0.429 | 0.909 |  |  |  |  |  |  |  |  |  |  |  |  |
| Smoker | 0.878 | 0.154 | 0.571 | 0.091 |  |  |  |  |  |  |  |  |  |  |  |  |
| **Sexual activity** |  |  |  |  |  |  |  |  |  |  |  |  |  |  |  |  |
| Never | 0.424 | 0.902 | 0.352 | 0.92 |  |  |  |  |  |  |  |  |  |  |  |  |
| Had intercourse | 0.576 | 0.098 | 0.648 | 0.08 |  |  |  |  |  |  |  |  |  |  |  |  |
| **Lifetime cannabis use** |  |  |  |  |  |  |  |  |  |  |  |  |  |  |  |  |
| Never | 0.553 | 0.986 | 0.663 | 0.995 |  |  |  |  |  |  |  |  |  |  |  |  |
| Any use | 0.447 | 0.014 | 0.337 | 0.005 |  |  |  |  |  |  |  |  |  |  |  |  |
| **Perceived academic achievement** |  |  |  |  |  |  |  |  |  |  |  |  |  |  |  |  |
| Very good | 0.025 | 0.047 | 0.102 | 0.157 |  |  |  |  |  |  |  |  |  |  |  |  |
| Good | 0.101 | 0.256 | 0.303 | 0.424 |  |  |  |  |  |  |  |  |  |  |  |  |
| Average | 0.622 | 0.625 | 0.517 | 0.41 |  |  |  |  |  |  |  |  |  |  |  |  |
| Below average | 0.252 | 0.072 | 0.077 | 0.009 |  |  |  |  |  |  |  |  |  |  |  |  |
| **Pressure from school work** |  |  |  |  |  |  |  |  |  |  |  |  |  |  |  |  |
| Not at all | 0.172 | 0.106 | 0.292 | 0.271 |  |  |  |  |  |  |  |  |  |  |  |  |
| A little | 0.429 | 0.602 | 0.482 | 0.595 |  |  |  |  |  |  |  |  |  |  |  |  |
| Some | 0.281 | 0.241 | 0.153 | 0.104 |  |  |  |  |  |  |  |  |  |  |  |  |
| A lot | 0.118 | 0.051 | 0.073 | 0.03 |  |  |  |  |  |  |  |  |  |  |  |  |
| **Classmate support scale** |  |  |  |  |  |  |  |  |  |  |  |  |  |  |  |  |
| High support | 0.208 | 0.205 | 0.39 | 0.477 |  |  |  |  |  |  |  |  |  |  |  |  |
| - | 0.172 | 0.177 | 0.194 | 0.228 |  |  |  |  |  |  |  |  |  |  |  |  |
| - | 0.288 | 0.36 | 0.231 | 0.21 |  |  |  |  |  |  |  |  |  |  |  |  |
| Low support | 0.332 | 0.258 | 0.185 | 0.084 |  |  |  |  |  |  |  |  |  |  |  |  |
| **Daily use of remote communication** |  |  |  |  |  |  |  |  |  |  |  |  |  |  |  |  |
| No daily use | 0.486 | 0.647 | 0.137 | 0.41 |  |  |  |  |  |  |  |  |  |  |  |  |
| Daily use | 0.514 | 0.353 | 0.863 | 0.59 |  |  |  |  |  |  |  |  |  |  |  |  |
| **Ease of communication with parents** |  |  |  |  |  |  |  |  |  |  |  |  |  |  |  |  |
| One easy to talk to | 0.399 | 0.351 | 0.606 | 0.612 |  |  |  |  |  |  |  |  |  |  |  |  |
| None easy to talk to | 0.601 | 0.649 | 0.394 | 0.388 |  |  |  |  |  |  |  |  |  |  |  |  |
| **Physical activity** |  |  |  |  |  |  |  |  |  |  |  |  |  |  |  |  |
| High | 0.095 | 0.075 | 0.316 | 0.147 |  |  |  |  |  |  |  |  |  |  |  |  |
| - | 0.097 | 0.15 | 0.257 | 0.221 |  |  |  |  |  |  |  |  |  |  |  |  |
| - | 0.286 | 0.291 | 0.27 | 0.39 |  |  |  |  |  |  |  |  |  |  |  |  |
| Low | 0.523 | 0.484 | 0.157 | 0.243 |  |  |  |  |  |  |  |  |  |  |  |  |
| **Fruit and vegetable consumption index** |  |  |  |  |  |  |  |  |  |  |  |  |  |  |  |  |
| High consumption | 0.086 | 0.062 | 0.251 | 0.217 |  |  |  |  |  |  |  |  |  |  |  |  |
| - | 0.136 | 0.148 | 0.221 | 0.273 |  |  |  |  |  |  |  |  |  |  |  |  |
| - | 0.244 | 0.283 | 0.282 | 0.311 |  |  |  |  |  |  |  |  |  |  |  |  |
| Low consumption | 0.534 | 0.507 | 0.246 | 0.199 |  |  |  |  |  |  |  |  |  |  |  |  |
| **Life satisfaction** |  |  |  |  |  |  |  |  |  |  |  |  |  |  |  |  |
| High | 0.082 | 0.045 | 0.356 | 0.334 |  |  |  |  |  |  |  |  |  |  |  |  |
| - | 0.15 | 0.166 | 0.303 | 0.378 |  |  |  |  |  |  |  |  |  |  |  |  |
| - | 0.207 | 0.239 | 0.211 | 0.208 |  |  |  |  |  |  |  |  |  |  |  |  |
| Low | 0.562 | 0.55 | 0.13 | 0.08 |  |  |  |  |  |  |  |  |  |  |  |  |
| **Finland** |  |  |  |  |  |  |  |  |  |  |  |  |  |  |  |  |
| **Weekly alcohol use** |  |  |  |  |  |  |  |  |  |  |  |  |  |  |  |  |
| Not weekly | 0.663 | 0.963 | 0.994 | 0.979 | 0.653 | 0.968 | 0.998 | 1 | 0.78 | 0.98 | 0.992 | 0.99 | 0.836 | 0.981 | 0.996 | 0.983 |
| Weekly | 0.337 | 0.037 | 0.006 | 0.021 | 0.347 | 0.032 | 0.002 | 0 | 0.22 | 0.02 | 0.008 | 0.01 | 0.164 | 0.019 | 0.004 | 0.017 |
| **Cigarette smoking** |  |  |  |  |  |  |  |  |  |  |  |  |  |  |  |  |
| Non-smoker | 0.113 | 0.825 | 0.599 | 0.843 | 0.134 | 0.82 | 0.847 | 0.905 | 0.174 | 0.842 | 0.843 | 0.905 | 0.329 | 0.919 | 0.925 | 0.972 |
| Smoker | 0.887 | 0.175 | 0.401 | 0.157 | 0.866 | 0.18 | 0.153 | 0.095 | 0.826 | 0.158 | 0.157 | 0.095 | 0.671 | 0.081 | 0.075 | 0.028 |
| **Sexual activity** |  |  |  |  |  |  |  |  |  |  |  |  |  |  |  |  |
| Never | 0.296 | 0.949 | 0.751 | 0.857 | 0.195 | 0.911 | 0.832 | 0.854 | 0.341 | 0.948 | 0.89 | 0.818 | 0.365 | 0.942 | 0.863 | 0.795 |
| Had intercourse | 0.704 | 0.051 | 0.249 | 0.143 | 0.805 | 0.089 | 0.168 | 0.146 | 0.659 | 0.052 | 0.11 | 0.182 | 0.635 | 0.058 | 0.137 | 0.205 |
| **Lifetime cannabis use** |  |  |  |  |  |  |  |  |  |  |  |  |  |  |  |  |
| Never | 0.654 | 0.986 | 0.964 | 0.989 | 0.691 | 0.986 | 1 | 0.989 | 0.615 | 0.972 | 1 | 0.968 | 0.628 | 0.984 | 0.988 | 0.972 |
| Any use | 0.346 | 0.014 | 0.036 | 0.011 | 0.309 | 0.014 | 0 | 0.011 | 0.385 | 0.028 | 0 | 0.032 | 0.372 | 0.016 | 0.012 | 0.028 |
| **Perceived academic achievement** |  |  |  |  |  |  |  |  |  |  |  |  |  |  |  |  |
| Very good | 0.045 | 0.103 | 0.157 | 0.308 | 0.055 | 0.131 | 0.199 | 0.271 | 0.059 | 0.09 | 0.292 | 0.333 | 0.037 | 0.116 | 0.283 | 0.392 |
| Good | 0.189 | 0.336 | 0.391 | 0.452 | 0.194 | 0.314 | 0.276 | 0.51 | 0.168 | 0.406 | 0.353 | 0.451 | 0.222 | 0.413 | 0.434 | 0.414 |
| Average | 0.602 | 0.502 | 0.41 | 0.24 | 0.557 | 0.515 | 0.449 | 0.211 | 0.545 | 0.414 | 0.338 | 0.202 | 0.519 | 0.438 | 0.272 | 0.178 |
| Below average | 0.164 | 0.059 | 0.042 | 0 | 0.194 | 0.039 | 0.076 | 0.008 | 0.228 | 0.09 | 0.017 | 0.015 | 0.222 | 0.032 | 0.011 | 0.015 |
| **Pressure from school work** |  |  |  |  |  |  |  |  |  |  |  |  |  |  |  |  |
| Not at all | 0.063 | 0.045 | 0.006 | 0.124 | 0.028 | 0.069 | 0.016 | 0.099 | 0.032 | 0.095 | 0.016 | 0.081 | 0.024 | 0.064 | 0.034 | 0.092 |
| A little | 0.325 | 0.494 | 0.404 | 0.565 | 0.343 | 0.553 | 0.276 | 0.572 | 0.249 | 0.371 | 0.207 | 0.491 | 0.251 | 0.46 | 0.248 | 0.464 |
| Some | 0.385 | 0.353 | 0.494 | 0.254 | 0.381 | 0.355 | 0.533 | 0.276 | 0.433 | 0.412 | 0.553 | 0.396 | 0.447 | 0.431 | 0.501 | 0.372 |
| A lot | 0.227 | 0.108 | 0.096 | 0.057 | 0.249 | 0.023 | 0.175 | 0.053 | 0.285 | 0.122 | 0.223 | 0.033 | 0.278 | 0.046 | 0.216 | 0.071 |
| **Classmate support scale** |  |  |  |  |  |  |  |  |  |  |  |  |  |  |  |  |
| High support | 0.221 | 0.134 | 0.085 | 0.384 | 0.22 | 0.143 | 0.064 | 0.306 | 0.206 | 0.182 | 0.139 | 0.435 | 0.266 | 0.235 | 0.187 | 0.49 |
| - | 0.326 | 0.371 | 0.157 | 0.317 | 0.3 | 0.394 | 0.169 | 0.319 | 0.286 | 0.325 | 0.207 | 0.31 | 0.278 | 0.409 | 0.228 | 0.312 |
| - | 0.238 | 0.313 | 0.4 | 0.202 | 0.302 | 0.286 | 0.327 | 0.251 | 0.236 | 0.329 | 0.317 | 0.149 | 0.223 | 0.244 | 0.355 | 0.121 |
| Low support | 0.215 | 0.182 | 0.358 | 0.096 | 0.178 | 0.177 | 0.44 | 0.123 | 0.273 | 0.163 | 0.337 | 0.106 | 0.233 | 0.112 | 0.229 | 0.078 |
| **Daily use of remote communication** |  |  |  |  |  |  |  |  |  |  |  |  |  |  |  |  |
| No daily use | 0.396 | 0.833 | 0.626 | 0.627 | 0.276 | 0.612 | 0.524 | 0.532 | 0.245 | 0.609 | 0.436 | 0.412 | 0.088 | 0.231 | 0.101 | 0.103 |
| Daily use | 0.604 | 0.167 | 0.374 | 0.373 | 0.724 | 0.388 | 0.476 | 0.468 | 0.755 | 0.391 | 0.564 | 0.588 | 0.912 | 0.769 | 0.899 | 0.897 |
| **Ease of communication with parents** |  |  |  |  |  |  |  |  |  |  |  |  |  |  |  |  |
| One easy to talk to | 0.325 | 0.211 | 0.121 | 0.491 | 0.33 | 0.227 | 0.128 | 0.461 | 0.294 | 0.281 | 0.203 | 0.522 | 0.384 | 0.302 | 0.189 | 0.614 |
| None easy to talk to | 0.675 | 0.789 | 0.879 | 0.509 | 0.67 | 0.773 | 0.872 | 0.539 | 0.706 | 0.719 | 0.797 | 0.478 | 0.616 | 0.698 | 0.811 | 0.386 |
| **Physical activity** |  |  |  |  |  |  |  |  |  |  |  |  |  |  |  |  |
| High | 0.064 | 0.048 | 0.028 | 0.124 | 0.126 | 0.087 | 0.047 | 0.195 | 0.147 | 0.079 | 0.092 | 0.239 | 0.18 | 0.088 | 0.112 | 0.292 |
| - | 0.149 | 0.17 | 0.12 | 0.286 | 0.258 | 0.206 | 0.201 | 0.406 | 0.184 | 0.2 | 0.281 | 0.385 | 0.228 | 0.21 | 0.277 | 0.37 |
| - | 0.367 | 0.299 | 0.424 | 0.353 | 0.295 | 0.34 | 0.392 | 0.292 | 0.336 | 0.356 | 0.393 | 0.295 | 0.341 | 0.414 | 0.397 | 0.265 |
| Low | 0.42 | 0.483 | 0.427 | 0.236 | 0.32 | 0.367 | 0.36 | 0.106 | 0.334 | 0.364 | 0.233 | 0.08 | 0.251 | 0.288 | 0.213 | 0.073 |
| **Fruit and vegetable consumption index** |  |  |  |  |  |  |  |  |  |  |  |  |  |  |  |  |
| High consumption | 0.079 | 0.024 | 0.214 | 0.202 | 0.101 | 0.02 | 0.163 | 0.277 | 0.076 | 0.008 | 0.275 | 0.237 | 0.065 | 0 | 0.233 | 0.221 |
| - | 0.163 | 0.096 | 0.234 | 0.348 | 0.191 | 0.079 | 0.258 | 0.307 | 0.172 | 0.09 | 0.276 | 0.348 | 0.146 | 0.066 | 0.329 | 0.371 |
| - | 0.291 | 0.374 | 0.333 | 0.27 | 0.33 | 0.304 | 0.298 | 0.349 | 0.359 | 0.343 | 0.309 | 0.332 | 0.407 | 0.358 | 0.333 | 0.356 |
| Low consumption | 0.467 | 0.506 | 0.219 | 0.181 | 0.378 | 0.597 | 0.28 | 0.066 | 0.392 | 0.559 | 0.14 | 0.082 | 0.383 | 0.577 | 0.105 | 0.052 |
| **Life satisfaction** |  |  |  |  |  |  |  |  |  |  |  |  |  |  |  |  |
| High | 0.237 | 0.132 | 0.044 | 0.687 | 0.217 | 0.222 | 0.114 | 0.579 | 0.146 | 0.22 | 0.16 | 0.631 | 0.162 | 0.237 | 0.098 | 0.625 |
| - | 0.325 | 0.459 | 0.342 | 0.277 | 0.289 | 0.394 | 0.299 | 0.366 | 0.331 | 0.363 | 0.313 | 0.35 | 0.249 | 0.426 | 0.336 | 0.3 |
| - | 0.205 | 0.233 | 0.244 | 0.034 | 0.229 | 0.261 | 0.209 | 0.054 | 0.179 | 0.254 | 0.259 | 0.019 | 0.246 | 0.198 | 0.248 | 0.076 |
| Low | 0.233 | 0.175 | 0.37 | 0.002 | 0.264 | 0.123 | 0.378 | 0 | 0.344 | 0.164 | 0.268 | 0 | 0.343 | 0.139 | 0.318 | 0 |

* 1: *Overall unhealthy. 2: Substance abstainers with behavioural risk indicators. 3: Moderately healthy. 4: Overall healthy.* ** Conditional response probabilities do not change over time for the Netherlands, Italy or Hungary; these results are therefore reported for one year only.
